# Factors associated with distinct prognostic‐awareness‐transition patterns over cancer patients’ last 6 months of life

**DOI:** 10.1002/cam4.4321

**Published:** 2021-09-30

**Authors:** Chen Hsiu Chen, Fur‐Hsing Wen, Wen‐Chi Chou, Jen‐Shi Chen, Wen‐Cheng Chang, Chia‐Hsun Hsieh, Siew Tzuh Tang

**Affiliations:** ^1^ School of Nursing National Taipei University of Nursing and Health Sciences Taipei Taiwan; ^2^ Department of International Business Soochow University Taipei Taiwan; ^3^ Division of Hematology‐Oncology Chang Gung Memorial Hospital Tao‐Yuan Taiwan; ^4^ College of Medicine Chang Gung University Tao‐Yuan Taiwan; ^5^ Department of Nursing Chang Gung Memorial Hospital at Kaohsiung Kaohsiung Taiwan; ^6^ School of Nursing Medical College Chang Gung University Tao‐Yuan Taiwan

**Keywords:** end‐of‐life care, neoplasms, oncology, prognostic awareness, transition patterns

## Abstract

**Background:**

Cancer patients may develop prognostic awareness (PA) heterogeneously, but predictors of PA‐transition patterns have never been studied. We aimed to identify transition patterns of PA and their associated factors during cancer patients’ last 6 months.

**Methods:**

For this secondary‐analysis study, PA was assessed among 334 cancer patients when they were first diagnosed as terminally ill and monthly till they died. PA was categorized into four states: (a) unknown and not wanting to know; (b) unknown but wanting to know; (c) inaccurate awareness; and (d) accurate awareness. The first and last PA states estimated by hidden Markov modeling were examined to identify their change patterns. Factors associated with distinct PA‐transition patterns were determined by multinomial logistic regressions focused on modifiable time‐varying variables assessed in the wave before the last PA assessment to ensure a clear time sequence for associating with PA‐transition patterns.

**Results:**

Four PA‐transition patterns were identified: maintaining accurate PA (56.3%), gaining accurate PA (20.4%), heterogeneous PA (7.8%), and still avoiding PA (15.6%). Reported physician‐prognostic disclosure increased the likelihood of belonging to the maintaining‐accurate‐PA group than to other groups. Greater symptom distress predisposed patients to be in the still‐avoiding‐PA than the heterogeneous PA group. Patients with higher functional dependence and more anxiety/depressive symptoms were more and less likely to be in the heterogeneous PA group and in the still‐avoiding‐PA group, respectively, than in the maintaining‐ and gaining‐accurate PA groups.

**Conclusions:**

Cancer patients heterogeneously experienced PA‐transition patterns over their last 6 months. Physicians’ prognostic disclosure, and patients’ symptom distress, functional dependence, and anxiety/depressive symptoms, all modifiable by high‐quality end‐of‐life care, were associated with distinct PA‐transition patterns.

## Abstract 2

Patients with cancer may not develop accurate prognostic awareness (PA) at the same rate or to the same degree and factors associated with PA‐transition patterns are unknown. Here, we identified PA‐transition patterns and their associated factors during the last six months of life for patients with advanced cancer and found that they developed PA in different ways. Four PA‐transition patterns were identified: maintaining accurate PA, gaining accurate PA, heterogeneous PA, and still avoiding PA. Prognostic disclosure by physicians, patient symptom distress, functional dependence, and anxiety/depression symptoms are major factors associated with PA‐transition patterns and can be acted on during high‐quality end‐of‐life care.

## 1 INTRODUCTION

Accurate prognostic awareness (PA) is essential for cancer patients to make value‐concordant end‐of‐life (EOL) care decisions,^1^ thus reducing potentially inappropriate life‐sustaining treatments (LSTs)^2^ to improve EOL‐care quality.^3^ However, approximately half of advanced cancer patients do not accurately understand their prognosis.^4^, ^5^ Implementing effective interventions to facilitate accurate PA requires understanding the underlying factors associated with cancer patients developing accurate PA.

Although factors associated with accurate PA for advanced cancer patients have been widely explored as highlighted in three systematic reviews,^6^, ^7^, ^8^ most studies were cross‐sectional, overlooking dynamic changes in patients’ PA. Patients’ PA changes over time as death approaches because physical condition deteriorates,^9^, ^10^ and physicians become more inclined to disclose prognosis.^11^ Indeed, our previous study^12^ showed that proportions of patients with accurate PA increased according to their proximity to death (from 61.7–62.9% to 68.3–76.1% from 151–180 to 1–30 days before death). However, cultivating accurate PA is a gradual process, especially for patients resistant to prognostic information, and reaching accurate PA near death does not leave sufficient time for patients to make important EOL decisions.^13^ Therefore, PA must be assessed throughout the course of the patient's disease.^13^ Furthermore, most cross‐sectional literature ignores directional relationships between PA and examined variables. Moreover, existing studies commonly neglect the heterogeneity of patients’ motivation for developing PA.^13^ Among the few studies exploring PA‐transition patterns,^14^, ^15^, ^16^, ^17^, ^18^, ^19^ only two^18^, ^19^ considered patients’ desire for prognostic information as a motivator for developing PA.^13^ Furthermore, factors associated with PA‐transition patterns have not yet been examined. Therefore, the purposes of this study were to identify PA‐transition patterns by considering motivation for PA during terminally ill cancer patients’ last 6 months and to examine factors associated with distinct PA‐transition patterns.

## 2 METHODS

### 2.1 Overview

Data for this secondary‐analysis study is from a blinded longitudinal randomized controlled trial of a tailored, multifaceted, interactive advance care planning intervention.^20^ Herein we explore patients’ PA‐transition patterns and factors associated with such patterns based on estimated PA states at first and last assessment within their last 6 months. Since experimental‐ and control‐arm prevalence of accurate PA did not differ at baseline and in patients’ last 60 days, PA data in both arms were combined.^12^

### 2.2 Setting and sample

Sampling details have been published.^20^ Briefly, consecutive adult cancer patients from a medical center in northwestern Taiwan were recruited when their oncologists first recognized their cancer as terminal (advanced disease continually progressed without responding to repeated chemotherapy/immunotherapy) from April 2013 to June 2017 and followed through September 2019. The study site ethics committee approved this study (101‐0898A3). All participants provided written informed consent.

## 3 MEASURES

### 3.1 Outcome variable: PA‐transition patterns

PA evaluations were based on Taiwanese physicians’ cultural practice of prognostic disclosure. Patients were asked if they knew their prognosis. If they did not know, subsequently they were asked to rate their desire for prognostic information (i.e., “Do you want your physician to tell you whether your disease can be cured or not?”) on a 5‐point Likert scale from 1 (“not desired at all”) to 5 (“very desired”). Desire for prognostic information was dichotomized at the median score into wanting to know (≥3) and not wanting to know (<3). If patients knew their prognosis, subsequently they were asked whether their disease (a) was curable; (b) might recur in the future, but their life was not currently in danger; or (c) could not be cured, and they would probably die in the near future.^21^ Patients were recognized as accurately knowing their PA if they chose option 3; inaccurate PA reflected not knowing their prognosis or choosing option 1 or 2.

Accordingly, patients’ PA was categorized into four states: (a) unknown and not wanting to know, (b) unknown but wanting to know, (c) inaccurate awareness, and (d) accurate awareness.^18^ PA states were estimated throughout patients’ last 6 months, and transition patterns in patients’ PA states were then identified based on the first and last estimated PA states^15^ during their last 6 months. (see *Data analysis*).

### 3.2 Independent variables

Based on Andersen's behavioral model,^22^ we hypothesized that changes in PA states during cancer patients’ last 6 months would be associated with (a) time‐invariant predisposing characteristics (e.g., demographics,^6^, ^7^ clinical characteristics,^6^, ^7^ and arms [experimental vs. control]); and time‐dependent variables, including (b) enabling resources (e.g., reported physicians’ prognostic disclosure^23^ and social support^7^); and (c) needs (e.g., patients’ disease burden^24^ and emotional distress).^17^

*Demographics and clinical characteristics*: Demographics included age, gender, marital status and educational level. Clinical characteristics were time since diagnosis and post‐enrollment survival.

*Reported physicians’ prognostic disclosure* was measured by asking patients whether their physician had disclosed their prognoses to them. Responses were coded yes (“1”) and no (“0”).

*Disease burden*: Physical symptoms and functional dependence were measured by the 13‐item Symptom Distress Scale (SDS)^25^ and 10‐item Enforced Social Dependency Scale (ESDS)^26^ respectively. The SDS is scored on a 5‐point Likert scale (1 [no distress]; 5 [extreme distress]).^25^ Total scores range from 13 to 65; higher scores indicate greater symptom distress. The ESDS assesses patients’ dependence on others’ assistance in executing daily activities; responses are rated on a 3‐ to 6‐point Likert scale.^26^ Total scores range from 10 to 51; higher scores reflect greater dependence on assistance for personal and social functioning.

*Emotional distress and social support*: Emotional distress and social support were measured by the 14‐item Hospital Anxiety and Depression Scale (HADS)^27^ and the Medical Outcomes Study Social Support Survey (MOS‐SSS)^28^ respectively. The HADS includes two subscales: depression (HADS‐D) and anxiety (HADS‐A). Each item is scored from 0 to 3 according to how respondents felt during the preceding week.^27^ Total subscale scores range from 0 to 21; higher scores indicate more severe depressive or anxiety symptoms. The 19‐item MOS‐SSS scale comprises five subscales: emotional, informational, tangible and affectionate support and positive social interaction. Each item is scored from 1 to 5.^28^ Raw scores are transformed to a total score from 0 to 100; higher scores indicate greater perceived social support.^28^

### 3.3 Data collection

Patients’ demographics and clinical characteristics were collected at enrollment. PA and time‐varying independent variables (e.g., physicians’ prognostic disclosure, physical symptom distress, functional dependence, anxiety symptoms, depressive symptoms and perceived social support) were collected by trained, experienced oncology nurses at enrollment and approximately monthly until patients declined participation or died.^20^

### 3.4 Data analysis

We used chi‐square tests and analysis of variance to compare baseline characteristics of patients in the final sample and those excluded from our analysis (Appendix S1 and Table S1). PA states and probabilities of shifting from one state to another between consecutive times (transition probability) were examined during patients’ last 6 months by a transition model with hidden Markov modeling (HMM) using Latent GOLD 5.0.^29^ For details on identifying distinct PA states and their transitions between consecutive times, see Appendix S2.

PA‐transition patterns were identified using the first and last PA states estimated by HMM. Factors associated with PA‐transition patterns were identified by multinomial logistic regressions. “Lagged measures” were used for time‐varying independent variables in the assessment wave before the last PA assessment to ensure a clear time sequence for associating with PA‐transition patterns. The regression estimate for each independent variable in the multinomial logistic regression models was exponentiated to transform into adjusted odds ratio (AOR) with 95% confidence interval (CI). *P* values ≤0.05 were statistically significant.

## 4 RESULTS

### 4.1 Sample characteristics

**FIGURE 1:**
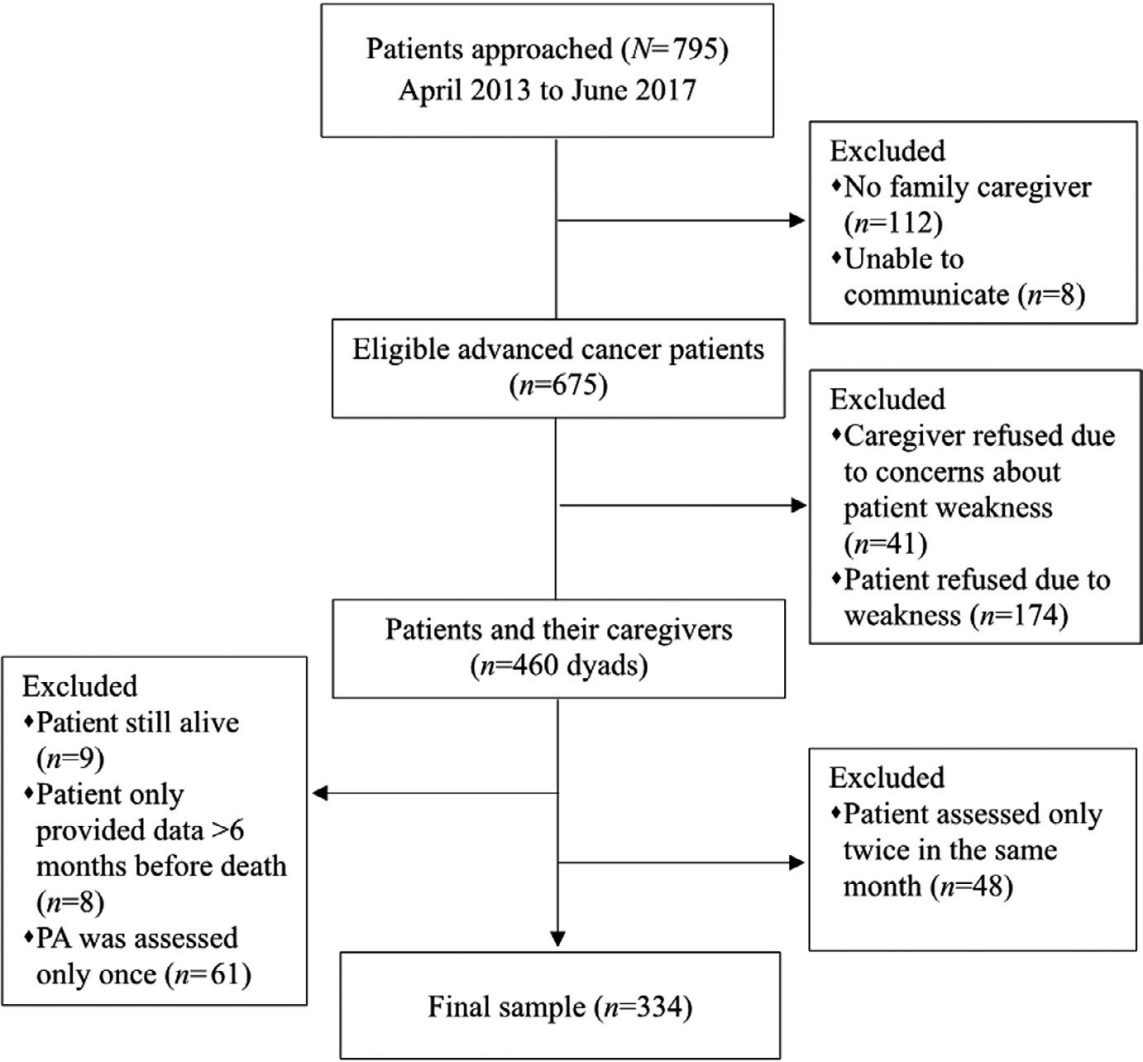
Participant flow chart

Of 795 eligible patients, 460 were enrolled (Figure 1). The 334 patients who died and supplied sufficient PA data for analyzing PA‐transition patterns comprised the final sample. Patients’ demographics, clinical characteristics, and time‐varying independent variables examined at the first assessment within their last 6 months are in Table S2. The first and last assessments were made, on average, 111.80 days (SD =51.28; median =125; range =31–183) and 23.69 days (SD =20.29; median =19; range =1–136) before death respectively.

**FIGURE 2:**
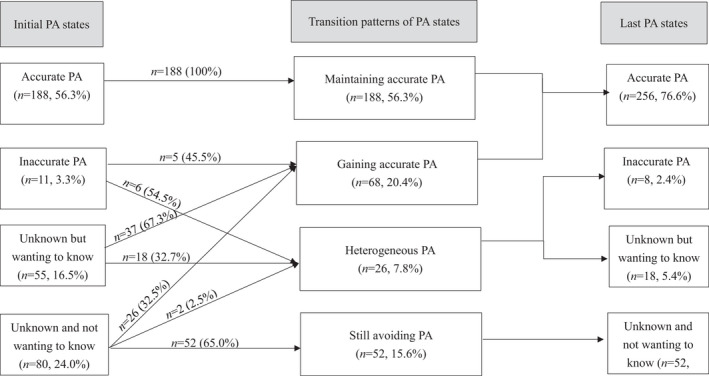
Longitudinal prognostic‐awareness‐transitional patterns between the first and last estimated state

Considering model‐fit indices (Appendix S3), parsimony, and the clinical meaningfulness of state identification, we identified four PA states. At baseline, accurate PA was the most prevalent PA state (*n *= 188, 56.3%), followed by unknown and not wanting to know (*n *= 80, 24.0%) and unknown but wanting to know (*n* = 55, 16.5%). Only 3.3% (*n* = 11) of participants had inaccurate PA (Figure 2).

Ranking of PA states was the same at last assessment based on HMM estimations (transition probability of PA states between consecutive times is in Appendix S4); however, the proportion of participants in each PA state changed: accurate PA (*n* = 256, 76.6%), unknown and not wanting to know (*n* = 52, 15.6%), unknown but wanting to know (*n*=18, 5.4%), and inaccurate PA (*n* = 8, 2.4%) (Figure 2).

### 4.2 PA‐transition patterns between the first and last estimations before death

Between the first and last assessments, all patients with accurate PA (*n* = 188, 100%) and approximately two‐thirds of those with unknown and not wanting to know PA (*n* = 52, 65.0%) remained in the same PA state (Figure 2). If patients’ PA state changed, it tended to shift toward the accurate‐PA state (gaining accurate PA), most likely for those who did not know but wanted to know their prognosis (*n* = 37, 67.3%), followed by those with inaccurate PA (*n* = 5, 45.5%) and those who did not know and did not want to know their prognosis (*n* = 26, 32.5%). Specifically, four PA‐transition patterns (prevalence) were identified: maintaining accurate PA (56.3%), gaining accurate PA (20.4%), heterogeneous PA (7.8%), and still avoiding PA (15.6%). The maintaining‐accurate‐PA group continuously had an accurate understanding of their prognosis. The gaining‐accurate‐PA group changed from not knowing their prognosis or from inaccurate PA to accurate PA. The heterogeneous‐PA group maintained inaccurate PA, maintained not knowing but wanting to know, or changed PA state from unknown and not wanting to know to inaccurate PA. Finally, the still‐avoiding‐PA group continuously did not know and did not want to know their prognosis.

### 4.3 Factors associated with PA‐transition patterns

For demographics and clinical characteristics, only age, educational level, and time from first interview to death were associated with the four PA‐transition patterns. The older the patients, the more likely they were to be in the still‐avoiding‐PA group than in the maintaining‐accurate‐PA group (AOR [95% CI]: 1.043 [1.005–1.082]) (Table 1). Patients with an education level ≥junior high school were less likely than those with less education to be in the still‐avoiding‐PA group than in the heterogeneous‐PA group (AOR [95% CI]: 0.293 [0.088–0.974]). Patients who survived longer post enrollment were less likely to be in the still‐avoiding‐PA group than in the maintaining‐ or gaining‐accurate‐PA groups (AOR [95% CI]: 0.992 [0.984–0.999] and 0.988 [0.980–0.996], respectively) as well as less likely to be in the heterogeneous‐PA group than in the gaining‐accurate‐PA group (AOR [95% CI]: 0.990 [0.980–0.999]).

**TABLE 1:** Factors associated with prognostic‐awareness transition states examined by multinomial logistic regressions (*N*=334)^a^.

Variable	Maintaining accurate PA^b^ versus						Gaining accurate PA^b^ versus				Heterogeneous PA^b^ versus	
	Gaining accurate PA		Heterogeneous PA		Still avoiding PA		Heterogeneous PA		Still avoiding PA		Still avoiding PA	
	AOR	95% CI	AOR	95% CI	AOR	95% CI	AOR	95% CI	AOR	95% CI	AOR	95% CI
Age	1.011	0.982–1.041	1.011	0.968–1.057	**1.043** *	**1.005–1.082**	1.000	0.955–1.048	1.031	0.992–1.072	1.031	0.981–1.083
Gender												
Female	1.178	0.590–2.352	2.768	0.998–7.678	1.671	0.707–3.949	2.350	0.794–6.952	1.419	0.580–3.473	0.604	0.195–1.865
Male	Ref		Ref		Ref		Ref		Ref		Ref	
Marital status												
Not married	2.006	0.845–4.759	1.176	0.303–4.567	1.132	0.360–3.560	0.586	0.148–2.327	0.564	0.184–1.731	0.963	0.227–4.083
Married	Ref		Ref		Ref		Ref		Ref		Ref	
Educational level												
>Junior high school	0.582	0.298–1.140	1.540	0.548–4.328	0.451	0.183–1.110	2.645	0.870–8.039	0.775	0.293–2.046	**0.293** *	**0.088–0.974**
<Junior high school	Ref		Ref		Ref		Ref		Ref		Ref	
Time since diagnosis	0.996	0.976–1.016	0.969	0.923–1.018	0.998	0.967–1.031	0.974	0.926–1.024	1.003	0.970–1.037	1.030	0.976–1.087
Time since the first interview to death^c^	1.004	0.997–1.010	0.994	0.985–1.003	**0.992** *	**0.984–0.999**	**0.990** *	**0.980–0.999**	**0.988** **	**0.980–0.996**	0.998	0.988–1.008
Prognostic disclosure												
Yes	**0.177** ***	**0.090–0.349**	**0.075** ***	**0.020–0.283**	**0.012** ***	**0.001–0.090**	0.425	0.101–1.786	**0.065** *	**0.008–0.536**	0.153	0.014–1.654
No	Ref		Ref		Ref		Ref		Ref		Ref	
Arm												
Experimental	1.015	0.542–1.903	0.530	0.204–1.378	1.010	0.467–2.184	0.522	0.188–1.450	0.995	0.441–2.246	1.905	0.662–5.487
Control	Ref		Ref		Ref		Ref		Ref		Ref	
SDS	1.033	0.977–1.093	0.952	0.871–1.039	1.066	0.992–1.146	0.921	0.839–1.012	1.032	0.956–1.114	**1.121** *	**1.016–1.236**
ESDS	0.974	0.928–1.022	**1.078** *	**1.004–1.157**	1.008	0.947–1.072	**1.107** **	**1.025–1.196**	1.035	0.969–1.105	0.935	0.862–1.014
HADS‐A	0.949	0.851–1.059	0.904	0.762–1.072	**0.865** *	**0.752–0.996**	0.950	0.794–1.142	0.911	0.786–1.058	0.957	0.791–1.159
HADS‐D	1.031	0.903–1.178	0.860	0.708–1.045	0.850	0.719–1.004	0.834	0.675–1.030	**0.824** *	**0.689–0.986**	0.988	0.795–1.228
MOS‐SSS	1.000	0.955–1.048	0.976	0.913–1.044	0.949	0.899–1.002	0.976	0.908–1.049	0.949	0.895–1.005	0.972	0.905–1.045

Note

Abbreviation: PA, prognostic awareness; SDS, Symptom Distress Scale; ESDS, Enforced Social Dependency Scale; HADS‐A, Hospital Anxiety and Depression Scale‐Anxiety; HADS‐D, Hospital Anxiety and Depression Scale‐Depression; MOS‐SSS, Medical Outcomes Study Social Support Survey; AOR, adjusted odds ratio; CI, confidence interval; Ref, reference; Bold indicates significance.

^a^ Three multinomial logistic regressions were performed, and different PA‐transition patterns as the reference group were indicated in each model.

^b^ Reference group.

^c^ In the last 6 months of life.

* *p* < 0.05

** *p* < 0.01

*** *p* < 0.001.

If patients reported that their physician had disclosed prognosis to them, they were more likely to be in the maintaining‐accurate‐PA group than in the other three groups (Table 1) as well as more likely to be in the gaining‐accurate‐PA group than in the still‐avoiding‐PA group. Patients with greater symptom distress were more likely to be in the still‐avoiding‐PA group (AOR [95% CI]: 1.121 [1.016–1.236]) than in the heterogeneous‐PA group. Patients with greater functional impairment were more likely to be in the heterogeneous‐PA group than in the maintaining‐ and gaining‐accurate‐PA group. Patients with more anxiety or depressive symptoms were less likely to be in the still‐avoiding‐PA group than in the maintaining‐accurate‐PA (AOR [95% CI]: 0.865 [0.752–0.996]) and the gaining‐accurate‐PA (AOR [95% CI]: 0.824 [0.689–0.986]) groups respectively.

## 5 DISCUSSION

We identified four PA‐transition patterns (prevalence): maintaining accurate PA (56.3%), gaining accurate PA (20.4%), heterogeneous PA (7.8%), and still avoiding PA (15.6%). Drawing from the transtheoretical model (TTM),^30^ patients with accurate PA are in the action/maintenance stages (e.g., receiving prognostic information and shifting to accurate PA) and are more likely to reinforce their PA than regress to less aware stages.^18^, ^30^ Indeed, we found that the PA status of all patients with accurate PA remained unchanged between the first and last assessments which may reflect reinforcement of patients’ accurate PA by deterioration of physical condition as death approaches.^18^, ^31^ Similarly, the TTM theorizes that people in the precontemplation stage (e.g., no desire for seeking prognostic information or not being ready to know one's prognosis [unknown and not wanting to know PA state]) do not intend to act in the foreseeable future.^18^, ^30^ Patients may not request prognostic information in fear of confronting bad news.^32^ Therefore, patients in the unknown and not wanting to know state at baseline had a high probability of still avoiding PA (65.0%). Conversely, patients with unknown but wanting to know (TTM contemplation stage [e.g., desire to or being ready to know one's prognosis]) and inaccurate PA (TTM preparation stage [e.g., ready to acquire prognostic information]) had a higher probability of shifting to accurate PA (45.5–67.3%) between the first and last assessments than of remaining in the original state (32.7–54.5%) (Figure 2), echoing a previous report.^18^

Our findings show that factors modifiable by high‐quality EOL care, that is, physicians’ prognostic disclosure, and patients’ symptom distress, functional dependence, anxiety symptoms, and depressive symptoms are associated with patients’ membership in the four distinct PA‐transition patterns. Reported physician‐prognostic disclosure predisposes patients to belong to the maintaining‐accurate‐PA group than to the other three groups. Indeed, patients whose physician had disclosed prognosis to^23^ or had discussed life expectancy with^16^ them were more likely to acknowledge being terminally ill. Therefore, for patients who are ready for prognostic communication, disclosing prognosis earlier and repeating it to cancer patients is important and necessary to consolidate and maintain their accurate PA, allowing them sufficient time to communicate their EOL‐care values and preferences to physicians and family caregivers^7^ and make informed EOL‐care decisions.^1^

Furthermore, patients who reported physicians disclosing prognosis to them were more likely to be in the gaining‐accurate‐PA group than in the still‐avoiding‐PA group. Our finding re‐confirms that physicians’ prognostic disclosure is the key to patients’ gaining accurate PA rather than remaining in the still‐avoiding‐PA group.^7^ However, the probability of reporting physician‐prognostic disclosure was similar for patients in the heterogeneous‐PA and the still‐avoiding‐PA groups. Our sample may be too small to detect a significant difference. If confirmed, this finding may reflect a missed opportunity for physicians to disclose prognosis to patients showing a strong motivation to know but lacking accurate PA (as in the heterogeneous‐PA group). Physicians should be sensitive to the prognostic‐information needs/desires of these patients and tailor interventions accordingly to cultivate accurate PA.

We found that patients with greater symptom distress were more likely to be in the still‐avoiding‐PA group than in the heterogeneous‐PA group. Furthermore, patients with greater functional impairment were more likely to be in the heterogeneous‐PA group than in the maintaining‐ and gaining‐accurate‐PA groups. Worse physical function/well‐being facilitates accurate PA^33^ because patients may want to know their prognosis if their physical condition deteriorates^31^ or they may guess their poor prognosis from their worsening physical function.^34^ However, we speculate that greater symptom distress and functional impairment draw physicians’ attention more to managing symptoms and facilitating physical functioning than to disclosing prognosis. Indeed, patients in the heterogeneous PA group were less likely to report physician‐prognostic disclosure than those in the maintaining and gaining accurate PA groups, whereas those in the still‐avoiding‐PA group were also less likely to report physician‐prognostic disclosure than those in the heterogeneous‐PA group (Table 1; note that some between‐group trends may not reach statistical significance due to insufficient power). Without physician‐prognostic disclosure, patients with more symptom distress and greater functional impairment may be more likely to belong to the heterogeneous‐PA or the still‐avoiding‐PA group than to the gaining‐accurate‐PA group. However, our speculation warrants validation, preferably by qualitative research.

In contrast, patients with more anxiety and depressive symptoms were less likely to belong to the still‐avoiding‐PA group than to the maintaining‐ or gaining‐accurate‐PA groups, respectively, as reported,^17^ but inconsistent with some literature.^35^, ^36^ Patients with more anxiety symptoms might be more motivated by their anxiety to acquire prognostic information and participate in prognostic discussions,^37^ thereby promoting their accurate PA. Patients with fewer depressive symptoms might not seek prognostic information to protect their emotional well‐being and maintain unrealistic hope,^17^ whereas those with more depressive symptoms might worry about their disease progressing and initiate prognostic discussions with their physicians, subsequently gaining accurate PA.^17^, ^38^ We used “lagged measures” for anxiety and depressive symptoms to establish a clear time sequence indicating emotional status driving PA‐transition patterns, rather than driving in the opposite direction.

Demographically, older patients were more likely to be in the still‐avoiding‐PA group than in the maintaining‐accurate‐PA group, as reported.^21^ In Taiwanese/Chinese culture, the principle of filial piety ensures that adult children are responsible for their parents’ well‐being^39^ and Taiwanese older people tend to rely on their adult children as medical surrogates.^40^ Adult children may protect their parents from adverse consequences associated with disclosing poor prognosis to them^41^ by asking physicians to conceal prognostic information from elderly parents, hindering them from gaining accurate PA. Healthcare professionals should not only explore elderly patients’ needs for prognostic information but also lessen family concerns about prognostic disclosure to facilitate accurate PA without damaging older patients’ psychological well‐being or hope.^42^ Furthermore, higher educational levels predisposed cancer patients to the heterogeneous‐PA group than in the still‐avoiding‐PA group, consistent with the literature.^43^

Clinically, patients who survived longer after their terminal status was first recognized by their oncologist were less likely to be in the heterogeneous‐PA and the still‐avoiding‐PA groups than in the maintaining‐ or gaining‐accurate‐PA groups. When cancer patients’ terminal/advanced status is recognized late, they may not have sufficient time for prognostic discussions and disclosure.^44^ We also found no between‐arm differences in the four distinct PA‐transition groups, thus further verifying our approach of combining experimental and control arms into one group based on our previous finding that experimental‐ and control‐arm prevalence of accurate PA did not differ at baseline and in patients’ last 60 days.^12^

### 5.1 Study limitations

Our study has several limitations. Generalization of our findings to national and international target populations may be limited because participants were sampled from a single medical center in Taiwan. Our findings should be replicated for advanced cancer patients in countries with different cultural, societal, and healthcare characteristics, especially around physicians’ prognostic disclosure. Furthermore, we excluded a noticeable proportion of patients from our analysis that did not provide sufficient monthly data for detecting PA‐transition patterns. We conducted a sensitivity test to include the 48 participants who were assessed only twice in a single month (Table S3) and found the results in line with our report except for the associations between PA‐transition groups and functional dependence and depressive symptoms, which may be due to small sample size in the heterogeneous PA group. Thus, generalizability of our findings may be limited to patients assessed only once or twice in the same month after enrollment, who may experience more depressive symptoms and have higher functional dependence. Recognizing terminal status by the referred oncologists may have questionable validity, but validated tools to recognize patients’ terminal status, such as the “surprise question,” are limited in Taiwan. PA‐transition patterns were analyzed based on only the first and last PA states estimated during patients’ last 6 months; thus, our findings may differ from PA‐transition patterns examined over patients’ entire last 6 months, but our approach is in line with the literature.^15^ Physicians’ prognostic disclosure was measured by patients’ retrospective reports without evaluating physicians’ perspective of their prognostic disclosure, presenting risk not only of recall bias but also of patients’ misunderstanding or misinterpreting physician's prognostic disclosure. Timing of assessments relative to study enrollment is various across all participants, but different lengths of post‐enrollment survival make equal time intervals between first and last PA assessment impossible. However, our approach of controlling lengths of survival not only post first diagnosis of cancer but also post first recognition of terminal status may provide a clear picture of where the participant was in their terminal trajectory. Given the small sample in each individual pattern, we condensed several PA‐transition patterns into the heterogeneous‐PA group, which may raise issues of heterogeneity in PA groups and complexity in detecting and explaining factors associated with PA‐transition patterns. To disentangle this heterogeneous PA‐transition pattern, a larger sample and different statistics may be needed to analyze and categorize PA‐transition patterns. Finally, we did not consider family caregivers’ perspectives, despite the well‐recognized relative power of Asian families in prognostic disclosure.^39^ The role of family caregivers in facilitating or impeding patients’ changes in PA warrants further investigation.

### 5.2 Clinical implications

Cancer patients experienced heterogeneous PA‐transition patterns over their last 6 months. Physicians’ prognostic disclosure, and patients’ symptom distress, functional dependence, anxiety symptoms, and depressive symptoms are major factors associated with patients’ membership in the distinct PA‐transition patterns and are modifiable through high‐quality EOL care. Physicians’ prognostic disclosure is the key to gaining and maintaining accurate PA. Therefore, physicians should be sensitive to patients’ prognostic‐information needs/desires and repeatedly initiate prognostic discussions earlier in the illness trajectory to facilitate/consolidate cancer patients accurate PA.

Healthcare professionals should not neglect the prognostic‐information needs of patients in the heterogeneous‐PA and still‐avoiding‐PA groups with greater symptom distress and functional dependence: to develop accurate PA these patients need adequate symptom management to relieve physical symptom distress and facilitate functioning, frequent assessment of their readiness for prognostic information, and prognostic discussions appropriately tailored to their prognostic‐information needs.

Furthermore, patients’ psychological distress (anxiety and depressive symptoms) may drive and facilitate accurate PA through worry about disease progression, earnestness to acquire prognostic information, and eager participation in prognostic discussions. Therefore, healthcare professionals should tailor prognostic discussions to the prognostic‐information needs of cancer patients with high anxiety and depressive symptoms and provide sufficient emotional support (i.e., inquiring about their feelings, attending to their emotions, offering ongoing support) with adequate referrals for psychological services after prognostic disclosure.^13^, ^32^ Furthermore, healthcare professionals not only should respect the self‐protection strategy of patients in the still‐avoiding‐PA group but also should carefully validate their emotional status,^17^ repeatedly assess their readiness for prognostic information, and tailor prognostic discussions to their needs. Through these actionable interventions, cancer patients may develop accurate PA earlier in their terminal‐illness trajectory and thereby make informed EOL‐care decisions^1^ to initiate high‐quality EOL care.^3^

## ETHICS STATEMENT

The institutional review board approval: 101‐0898A3.

## CONFLICT OF INTEREST

The authors declare no financial or other conflict of interest.

No funding sources had any role in designing and conducting the study; collecting, managing, analyzing, and interpreting the data; or preparing, reviewing, or approving the article.

## Supporting information

Supplementary Material

## Data Availability

The sharing of anonymized data from this study is restricted due to ethical and legal constrictions. Data contains sensitive personal health information, which is protected under The Personal Data Protection Act, thus making all data requests subject to Institutional Review Board (IRB) approval. Per Chang Gung Memorial Hospital (CGMH) IRB, the data that support the findings of this study are restricted for transmission to those within the primary investigative team. Data sharing with investigators outside the team requires IRB approval. All requests for anonymized data will be reviewed by the research team and then submitted to the CGMH IRB for approval.
