# Supplementary material for: Factors associated with distinct prognostic‐awareness‐transition patterns over cancer patients’ last 6 months of life
Source: Cancer Med. 2021 Sep 30;10(22):8029–39. doi: 10.1002/cam4.4321 (PMC8607263; doi:10.1002/cam4.4321)
Supplement: Supplementary file 1 — Supplementary Material [file CAM4-10-8029-s001.docx]

**Appendix 1.** Description of participants excluded and results of the aforementioned comparisons.

Participants were excluded if (1) their PA data were collected only >6 months before death (thus avoiding spurious data from the few patients who survived >6 months), they were (2) assessed only once (at least two assessments are required to analyze PA-transition patterns), (3) assessed only twice, both times in the same month (since patients’ PA states were estimated every month), or (4) still alive at study end.

These participants and those who were excluded (Appendix 1) did not differ significantly in baseline demographics, clinical characteristics, and PA, except for disease burden, and emotional distress (Supplemental Table 1). Patients whose PA was assessed only >6 months before death experienced lower symptom distress than study patients. In addition, patients whose PA was assessed only once, or twice but in the same month, reported greater symptom distress, functional dependence, and depressive symptoms than study patients.

**Appendix 2**. Transition model with hidden Markov modeling to identify optimal number of distinct prognostic awareness (PA) states and to estimate transition probabilities

In the first part of hidden Markov modeling (HMM),^1^ patients were allocated based on their shared characteristics to a confined number of mutually exclusive probabilistic PA states each month. The identified states, which cannot be observed directly, are latent and estimated. Therefore, the distribution function for response variables in each state reflects the observed probability that each patient would not know and not want to know, not know but want to know, have inaccurate awareness, or have accurate awareness.^2^

Models were evaluated by model choice for the optimal number of PA transitional states, the Akaike information criterion (AIC),^3^ the Bayesian information criterion (BIC),^4^ the consistent AIC (CAIC),^5^ sample size-adjusted BIC (SABIC),^6^ and log-likelihood (LL).^7^ Generally, lower AIC, BIC, and CAIC but higher LL indicate a better model-data fit. These criteria and clinical meaningfulness of latent class results were used to determine the optimal number of states.

In the second part of HMM, PA state-transition probabilities were estimated.^1^ Transition probability represents the likelihood that a patient would stay in a specific PA state at time t, given his/her PA state at time t-1. Based on each state’s initial probability (size) and transition probabilities, we estimated each participant’s PA state before death.

**References**:

1. Vermunt JK, Tran B, Magidson J. Latent class models in longitudinal research. In: Menard S, ed. *Handbook of Longitudinal Research: Design, Measurement, And Analysis*. Burlington, MA: Elsevier; 2008:373-385.

2. Eddy SR. Profile hidden Markov models. *Bioinformatics.* 1998;14(9):755-763.

3. Akaike H. Factor analysis and AIC. *Psychometrika*. 1987;52(3):317-332.

4. Schwarz G. Estimating the dimension of a model. *Ann Stat*. 1978;6(2):461-464.

5. Bozdogan H. Model selection and Akaike’s information criterion (AIC): the general theory and its analytical extensions. *Psychometrika*. 1987;52(3):345-370.

6. Sclove LS. Application of a model-selection criteria to some problems in multivariate analysis. *Psychometrika*. 1987;52(3):333-343.

7. Nylund KL, Asparouhov T, Muthén BO. Deciding on the number of classes in latent class analysis and growth mixture modeling: a Monte Carlo simulation study. *Struct Equ Model*. 2007;14(4):535-569.

**Appendix** **3**. Measurement of model-fit statistics for one- to six-state solutions of PA

| State number | LL | BIC(LL) | AIC(LL) | CAIC(LL) | SABIC(LL) |
| --- | --- | --- | --- | --- | --- |
| 1 | -1228.01 | 2473.85 | 2462.01 | 2476.85 | 2464.33 |
| 2 | -946.10 | 1945.71 | 1910.20 | 1954.71 | 1917.15 |
| 3 | -835.02 | 1771.11 | 1704.03 | 1788.11 | 1717.17 |
| **4** | -794.06 | **1748.65** | **1642.13** | **1775.65** | **1662.99** |
| 5 | -786.47 | 1804.81 | 1650.94 | 1843.81 | 1681.07 |
| 6 | **-782.68** | 1880.47 | 1671.36 | 1933.47 | 1712.31 |

Abbreviation: LL, log-likelihood; BIC, the Bayesian information criterion; AIC, the Akaike information criterion; CAIC, the consistent AIC; SABIC, sample size-adjusted BIC.

Bold indicates the optimal number of states based on the criteria and clinical meaningfulness of latent class results.

**Appendix 4**. Transition probabilities of prognostic awareness from time point [t-1] to time point [t] among cancer patients in their last 6 months

| State of Prognostic Awareness | Time Point [t] | | | |
| --- | --- | --- | --- | --- |
| Time Point [t-1] | Unknown and not wanting to know | Unknown but wanting to know | Inaccurate PA | Accurate PA |
| Unknown and not wanting to know | **0.894** | 0.000 | 0.013 | 0.093 |
| Unknown but wanting to know | 0.037 | **0.816** | 0.000 | 0.147 |
| Inaccurate PA | 0.055 | 0.039 | **0.815** | 0.091 |
| Accurate PA | 0.000 | 0.000 | 0.000 | **1.000** |

Bold indicates the highest transition probability between time points.

**Supplemental Table 1.** Baseline characteristics of final sample and excluded participants

| Variable | Final sample  (*N*=334) | Alive  (*n*=9) | PA provided only  >6 months  before death  (*n=*8) | PA assessed only once  (*n=*61) | PA assessed only twice in same month  (*n*=48) |
| --- | --- | --- | --- | --- | --- |
| Age, years  Mean (SD) | 57.93 (11.15) | 55.00 (10.21) | 55.60  (16.79) | 58.27  (11.41) | 57.73  (10.89) |
| Gender, No. (%) |  |  |  |  |  |
| Male | 234  (70.1) | 6  (66.7) | 7  (7.8) | 43  (72.9) | 33  (68.8) |
| Female | 100  (29.9) | 3  (33.3) | 3  (22.2) | 16  (27.1) | 15  (31.3) |
| Marital status, No. (%) | |  |  |  |  |
| Married | 284  (85.0) | 8  (88.9) | 8  (80.0) | 49  (83.1) | 43  (89.6) |
| Unmarried | 50  (15.0) | 1  (11.1) | 2  (20.0) | 10  (16.9) | 5  (10.4) |
| Educational level, No. (%) | |  |  |  |  |
| >Junior high school | 135  (40.5) | 3  (33.3) | 6  (60.0) | 24  (40.7) | 16  (33.3) |
| ≤Junior high school | 198  (59.5) | 6  (66.7) | 4  (40.0) | 35  (59.3) | 32  (66.7) |
| Financial status, No. (%) | |  |  |  |  |
| Sufficient | 247  (87.0) | 7  (77.8) | 8  (80.0) | 46  (88.5) | 36  (83.7) |
| Insufficient | 37  (13.0) | 2  (22.2) | 2  (20.0) | 6  (11.5) | 7  (16.3) |
| Cancer site, No. (%) |  |  |  |  |  |
| Liver | 69  (20.7) | 4  (44.4) | 3  (30.0) | 11  (18.6) | 13  (27.1) |
| Stomach-intestine | 61  (18.3) | 0  (0.0) | 1  (10.0) | 17  (28.8) | 14  (29.2) |
| Esophagus | 61  (18.3) | 2  (22.2) | 0  (0.0) | 8  (13.6) | 2  (4.2) |
| Pancreas | 51  (15.4) | 1  (11.1) | 0  (0.0) | 10  (16.9) | 6  (12.5) |
| Other | 92  (27.6) | 2  (22.2) | 6  (60.0) | 13  (22.0) | 13  (27.1) |
| Metastasis, No. (%) |  |  |  |  |  |
| Yes | 315  (94.3) | 8  (88.9) | 9  (90.0) | 56  (94.9) | 47  (97.9) |
| No | 19  (5.7) | 1  (11.1) | 1  (10.0) | 3  (5.1) | 1  (2.1) |
| Chronic disease, No. (%) | |  |  |  |  |
| Yes | 223  (66.8) | 8  (88.9) | 6  (60.0) | 39  (66.1) | 35  (72.9) |
| No | 111  (33.2) | 1  (11.1) | 4  (40.0) | 20  (33.9) | 13  (27.1) |
| Post-diagnostic survival, months | | |  |  |  |
| Mean (SD) | 11.30 (15.13) | 11.11 (13.47) | 12.30  (9.01) | 9.80  (12.76) | 11.98  (13.89) |
| Prognostic disclosure, No. (%) | |  |  |  |  |
| Yes | (29.2) | (22.2) | (40.0) | (32.2) | (41.7) |
| No | (70.8) | (77.8) | (60.0) | (67.8) | (58.3) ) |
| Prognostic awareness, No. (%) | |  |  |  |  |
| Accurate PA | 176  (52.7) | 4  (44.4) | 5  (50.0) | 37  (62.7) | 27  (56.3) |
| Inaccurate PA | 19  (5.7) | 1  (11.1) | 1  (10.0) | 1  (1.7) | 3  (6.6) |
| Unknown but wanting to know | 61  (18.3) | 2  (22.2) | 2  (20.0) | 10  (16.9) | 7  (14.6) |
| Unknown and not wanting to know | 78  (23.4) | 2  (22.2) | 2  (20.0) | 11  (18.6) | 11  (22.9) |
| SDS score,  Mean (SD) | 25.27 (5.87) | 24.22  (4.21) | 21.00^*^  (5.21) | 31.35^**^  (8.02) | 31.26^**^  (7.51) |
| ESDS score,  Mean (SD) | 26.13 (8.10) | 28.67  (10.27) | 25.70  (8.21) | 32.14^**^  (9.98) | 32.73^**^  (7.87) |
| HADS-A score,  Mean (SD) | 5.91 (3.63) | 7.00  (3.12) | 6.50  (3.14) | 6.93  (4.34) | 7.03  (3.78) |
| HADS-D score,  Mean (SD) | 10.85 (4.37) | 11.11  (4.34) | 10.00  (2.75) | 13.58^**^  (4.25) | 13.69^**^  (4.03) |
| MOS-SSS score,  Mean (SD) | 64.91 (9.21) | 62.30  (10.67) | 62.70  (8.93) | 62.30  (8.85) | 62.66  (8.41) |

Abbreviation: PA, prognostic awareness; SDS, Symptom Distress Scale; ESDS, Enforced Social Dependency Scale; HADS-A, Hospital Anxiety and Depression Scale-Anxiety; HADS-D, Hospital Anxiety and Depression Scale-Depression; MOS-SSS, Medical Outcomes Study Social Support Survey.

^*^*P* <.05; ^**^ *P* <.001

**Supplemental Table 2**. participant demographics and clinical characteristics

| Variable ^†^ |  | Prognostic awareness transition patterns | | | |
| --- | --- | --- | --- | --- | --- |
|  | Total  (*N*=334) | Maintaining accurate PA  (*n*=188) | Gaining accurate PA  (*n*=68) | Heterogeneous PA  (*n*=26) | Still avoiding PA  (*n*=52) |
| Age, years  Mean (SD) | 57.93 (11.15) | 56.36 (10.40) | 58.24  (11.68) | 58.58  (13.23) | 62.87  (10.73) |
| Gender, No. (%) |  |  |  |  |  |
| Male | 234  (70.1) | 139  (73.9) | 46  (67.6) | 16  (61.5) | 33  (63.5) |
| Female | 100  (29.9) | 49  (26.1) | 22  (32.4) | 10  (38.5) | 19  (36.5) |
| Marital status, No. (%) | |  |  |  |  |
| Married | 284  (85.0) | 164  (87.2) | 55  (80.9) | 22  (84.6) | 43  (82.7) |
| Not married | 50  (15.0) | 24  (12.8) | 13  (19.1) | 4  (15.4) | 9  (17.3) |
| Educational level, No. (%) | |  |  |  |  |
| >Junior high school | 135  (40.5) | 90  (48.1) | 21  (30.9) | 12  (46.2) | 12  (23.1) |
| ≤Junior high school | 198  (59.5) | 97  (51.9) | 47  (69.1) | 14  (53.8) | 40  (76.9) |
| Financial status, No. (%) | |  |  |  |  |
| Sufficient | 247  (87.0) | 133  (87.5) | 56  (87.5) | 19  (82.6) | 39  (86.7) |
| Insufficient | 37  (13.0) | 19  (12.5) | 8  (12.5) | 4  (17.4) | 6  (13.3) |
| Cancer site, No. (%) |  |  |  |  |  |
| Liver | 69  (20.7) | 48  (25.5) | 7  (10.3) | 2  (7.7) | 12  (23.1) |
| Stomach-intestine | 61  (18.3) | 31  (16.5) | 17  (25.0) | 6  (23.1) | 7  (13.5) |
| Esophagus | 61  (18.3) | 31  (16.5) | 12  (17.8) | 6  (23.1) | 12  (23.1) |
| Pancreas | 51  (15.4) | 32  (17.0) | 12  (17.6) | 2  (7.7) | 5  (9.6) |
| Other | 92  (27.6) | 41  (24.5) | 18  (29.4) | 2  (38.5) | 9  (30.8) |
| Metastasis, No. (%) |  |  |  |  |  |
| Yes | 315  (94.3) | 178  (94.7) | 66  (97.1) | 24  (92.3) | 47  (90.4) |
| No | 19  (5.7) | 10  (5.3) | 2  (2.9) | 2  (7.7) | 5  (9.6) |
| Chronic disease, No. (%) | |  |  |  |  |
| Yes | 223  (66.8) | 128  (68.1) | 45  (66.2) | 19  (73.1) | 31  (59.6) |
| No | 111  (33.2) | 60  (31.9) | 23  (33.8) | 7  (26.9) | 21  (40.4) |
| Post-diagnostic survival, months | |  |  |  |  |
| Mean (SD) | 11.30 (15.13) | 12.58 (13.77) | 10.99 (21.55) | 7.54  (10.22) | 8.94  (11.09) |
| Median (range) | 6  (0-121) | 9  (0-87) | 4  (0-121) | 3  (0-40) | 6  (0-48) |
| Post-enrollment survival, days^‡^ | |  |  |  |  |
| Mean (SD) | 111.80 (51.28) | 113.94  (50.71) | 123.56  (49.97) | 95.42  (51.73) | 96.85  (50.47) |
| Median (range) | 124  (31-183) | 125  (31-183) | 152  (32-180) | 79  (31-173) | 84  (31-176) |
| Prognostic disclosure, No. (%) | |  |  |  |  |
| Yes | 113  (34.1) | 109  (58.0) | 3  (4.5) | 1  (3.8) | 0  (0.0) |
| No | 218  (65.9) | 79  (42.0) | 64  (95.5) | 25  (96.2) | 50  (100.0) |
| SDS score,  Mean (SD) | 26.84 (6.76) | 27.16  (7.05) | 25.62  (6.60) | 25.58  (6.58) | 27.94  (5.80) |
| ESDS score,  Mean (SD) | 26.04 (7.97) | 25.92  (7.70) | 23.65  (7.45) | 28.12  (8.71) | 28.56  (8.41) |

| Variable ^†^ |  | Prognostic awareness transition patterns | | | |
| --- | --- | --- | --- | --- | --- |
|  | Total  (*N*=334) | Maintaining accurate PA  (*n*=188) | Gaining accurate PA  (*n*=68) | Heterogeneous PA  (*n*=26) | Still avoiding PA  (*n*=52) |
| HADS-A score,  Mean (SD) | 5.86 (3.55) | 6.09  (3.71) | 5.82  (3.58) | 5.42  (3.15) | 5.35  (3.06) |
| HADS-D score,  Mean (SD) | 11.21 (4.40) | 11.19  (4.53) | 10.59  (4.23) | 10.81  (4.45) | 12.27  (4.06) |
| MOS-SSS score,  Mean (SD) | 64.80 (8.49) | 65.50  (8.41) | 65.16  (8.75) | 64.28  (5.62) | 62.09  (9.21) |

Abbreviation: SDS, Symptom Distress Scale; ESDS, Enforced Social Dependency Scale; HADS-A, Hospital Anxiety and Depression Scale-Anxiety; HADS-D, Hospital Anxiety and Depression Scale-Depression; MOS-SSS, Medical Outcome Study Social Support Survey

^†^ Time-varying variables in the first assessed within participants’ last 6 months.

^‡^ In the last six months of life.

**Supplemental Table 3**. Sensitivity test for factors associated with prognostic-awareness transition states examined by multinomial logistic regressions (*N*=382) ^†^

|  | Maintaining accurate PA^‡^ vs. | | | | | | Gaining accurate PA^‡^ vs. | | | | Heterogeneous PA ^‡^ vs. | |
| --- | --- | --- | --- | --- | --- | --- | --- | --- | --- | --- | --- | --- |
| Variable | Gaining accurate PA | | Heterogeneous PA | | Still avoiding PA | | Heterogeneous PA | | Still avoiding PA | | Still avoiding PA | |
|  | AOR | 95% CI | AOR | 95% CI | AOR | 95% CI | AOR | 95% CI | AOR | 95% CI | AOR | 95% CI |
| Age | 1.004 | 0.977-1.033 | 1.022 | 0.981-1.064 | **1.035**^*^ | **1.000-1.072** | 1.017 | 0.973-1.063 | 1.031 | 0.993-1.070 | 1.014 | 0.968-1.061 |
| Gender |  |  |  |  |  |  |  |  |  |  |  |  |
| Female | 1.046 | 0.531-2.061 | 1.806 | 0.682-4.710 | 1.857 | 0.846-4.077 | 1.726 | 0.614-4.852 | 1.775 | 0.766-4.114 | 1.028 | 0.362-2.922 |
| Male | Ref |  | Ref |  | Ref |  | Ref |  | Ref |  | Ref |  |
| Marital status |  |  |  |  |  |  |  |  |  |  |  |  |
| Not married | 1.984 | 0.849-4.635 | 1.385 | 0.389-4.933 | 1.137 | 0.380-3.403 | 0.698 | 0.188-2.595 | 0.573 | 0.193-1.704 | 0.821 | 0.206-3.271 |
| Married | Ref |  | Ref |  | Ref |  | Ref |  | Ref |  | Ref |  |
| Educational level |  |  |  |  |  |  |  |  |  |  |  |  |
| > Junior high school | 0.552 | 0.286-1.068 | 1.403 | 0.544-3.618 | 0.514 | 0.224-1.181 | 2.540 | 0.899-7.179 | 0.931 | 0.373-2.324 | **0.367**^*^ | **0.121-0.998** |
| <Junior high school | Ref |  | Ref |  | Ref |  | Ref |  | Ref |  | Ref |  |
| Time since diagnosis | 0.992 | 0.972-1.012 | 0.972 | 0.934-1.011 | 0.984 | 0.955-1.013 | 0.980 | 0.940-1.022 | 0.992 | 0.961-1.024 | 1.012 | 0.968-1.059 |
| Time since the first interview to death^§^ | 1.005 | 0.999-1.011 | 0.995 | 0.987-1.003 | **0.992**^*^ | **0.986-0.999** | **0.990**^*^ | **0.982-0.999** | **0.987**^**^ | **0.980-0.995** | 0.997 | 0.988-1.006 |
| Prognostic disclosure |  |  |  |  |  |  |  |  |  |  |  |  |
| Yes | **0.157**^***^ | **0.081-0.308** | **0.103**^***^ | **0.033-0.321** | **0.010**^***^ | **0.001-0.079** | 0.656 | 0.186-2.313 | **0.066**^*^ | **0.008-0.532** | 0.100 | 0.010-1.001 |
| No | Ref |  | Ref |  | Ref |  | Ref |  | Ref |  | Ref |  |
| Arm |  |  |  |  |  |  |  |  |  |  |  |  |
| Experimental | 1.043 | 0.569-1.914 | 0.631 | 0.265-1.507 | 0.907 | 0.444-1.855 | 0.605 | 0.236-1.555 | 0.869 | 0.402-1.879 | 1.437 | 0.545-3.785 |
| Control | Ref |  | Ref |  | Ref |  | Ref |  | Ref |  | Ref |  |
| SDS | 1.028 | 0.974-1.085 | 0.945 | 0.873-1.023 | 1.047 | 0.980-1.118 | 0.920 | 0.844-1.002 | 1.019 | 0.949-1.093 | **1.107**^*^ | **1.014-1.209** |
| ESDS | 0.974 | 0.930-1.020 | 1.032 | 0.968-1.100 | 1.002 | 0.948-1.058 | 1.060 | 0.988-1.137 | 1.029 | 0.969-1.092 | 0.971 | 0.904-1.043 |
| HADS-A | 0.943 | 0.849-1.048 | 0.942 | 0.809-1.097 | **0.873**^*^ | **0.769-0.992** | 0.999 | 0.846-1.179 | 0.926 | 0.807-1.063 | 0.927 | 0.780-1.100 |
| HADS-D | 1.052 | 0.930-1.189 | 0.958 | 0.813-1.128 | 0.907 | 0.785-1.047 | 0.911 | 0.759-1.093 | 0.862 | 0.735-1.001 | 0.946 | 0.785-1.141 |
| MOS-SSS | 1.001 | 0.958-1.045 | 0.998 | 0.941-1.059 | 0.958 | 0.913-1.005 | 0.998 | 0.935-1.065 | 0.949 | 0.895-1.005 | 0.959 | 0.900-1.023 |

Abbreviation: PA, prognostic awareness; SDS, Symptom Distress Scale; ESDS, Enforced Social Dependency Scale; HADS-A, Hospital Anxiety and Depression Scale-Anxiety; HADS-D, Hospital Anxiety and Depression Scale-Depression; MOS-SSS, Medical Outcomes Study Social Support Survey; AOR, adjusted odds ratio; CI, confidence interval; Ref, reference; Bold indicates significance.

^*^*P* <.05; ^**^ *P* <.01; ^***^ *P* <.001

^†^ Three multinomial logistic regressions were performed, and different PA-transition patterns as the reference group were indicated in each model.

^‡^ Reference group.

^§^ In the last six months of life.
